# The Effect of Age on the Organs of the Horse

**Published:** 1894-12

**Authors:** J. T. Duncan

**Affiliations:** Professor of Anatomy, Ontario Veterinary College, Toronto


### THE JOURNAL

OF

### COMPARATIVE MEDICINE AND VETERINARY ARCHIVES.

Vol. XV. DECEMBER, 1894. No. 7.

### THE EFFECT OF AGE ON THE ORGANS OF THE HORSE.

By J. T. Duncan,

*Professor of Anatomy, Ontario Veterinary College, Toronto.*

In the dissecting room of the Ontario Veterinary College it has been our custom for some time back carefully to weigh each seperate organ of every subject. A printed form is issued with spaces for entering such weights, as well as other particulars. As dissection proceeds the weights are entered in their proper spaces by the Secretary. When the subject is finished the filled in form is returned to the college and kept for future examination. At the close of the session the averages are made up. In this way we have the weights of the right and left lungs, heart, liver, spleen, pancreas, right and left kidneys and brain. By this plan not only are habits of exactitude fostered among the students, and the memory aided in regard to the weights of the organs, but we also have interesting data to compare with those given in the standard text-books.

I have only as yet made up the returns from a portion of the work of two sessions. The cases however have not been selected, they are consecutive, and up to date.

In order the better to compare our results with those from standard works I shall place them in parallel columns. Those in the first column are from that admirable French work (translated by Dr. G. Fleming), viz;—Chauveau's Comparative Anatomy of the Domesticated Animals. The column showing the number of subjects, and the last column of weights, are from the returns of the Ontario Veterinary College.

| | CHAUVEAU. | | | O. V. COLLEGE. | | | |
|---|---|---|---|---|---|---|---|
| | lbs. | oz. | dr. | No. of Subjects. | lbs. | oz. | dr. |
| Heart, | 6 | 12 | .. | 89 | 7 | 3 | .. |
| Liver, | 11 | .. | .. | 92 | 9 | 7 | .. |
| Spleen, | .. | 32 | .. | 89 | .. | 34 | 8 |
| Pancreas, | .. | 17 | .. | 78 | .. | 14 | 12 |
| Right Kidney, | .. | 27 | .. | 87 | .. | 24 | 8 |
| Left Kidney, | .. | 25 | .. | 86 | .. | 22 | 4 |
| Brain, | .. | 22 | 15 | 74 | .. | 23 | 4 |

Chauveau's work gives no idea of the number of horses examined, but makes it clearly understood that the weights published are from healthy, middle sized, adult horses.

On the other hand it must be remembered that the returns from this College are from dissecting room subjects.

Horses sent to the dissecting room are as a rule aged animals. Chauveau's figures, then, are from full grown but not aged subjects. Ours, on the contrary, are from old and generally worn out animals. As to size ours were probably an average lot.

What effect upon the weight of the different organs would the discrepancy in age be likely to produce? Speaking generally it may be said that the organs atrophy with age. The heart is an exception to this rule. Figures from this college, then, should show all the organs to be lighter than those given in anatomical works, except the heart. Taking the organs seperately we have for consideration ;—

1. *The Heart.* This organ in the College series weighs seven ounces (nearly half a pound) heavier than Chauveau's figures give. This is not surprising as the tendency of the heart is not towards atrophy but rather to increase both in bulk and weight as age advances.

2. *Liver.* This gland at the second month of uterine life constitutes about one-half of the entire weight of the body, at birth it is about 1 to 18, while in the adult it is not more than 1 to 40. These figures refer to the human being, but the discrepancy is even greater in the horse. In the adult horse the proportion is about 1 to 80 of the weight of the animal. These figures emphasize what has already been stated as a general law, viz., that age causes atrophy. By referring to our weights, it will be seen that the liver of the aged horse is 1 lb. 9 ozs. lighter than that of the adult.

3. *Spleen.* The tendency of this organ is very decidedly to atrophy with age, yet we find our figures show the spleen of the old animal to be 2½ ozs. heavier than that of the adult.

To explain this is not easy. It may be due to accident, thus, if one case of considerable hypertrophy were to be found among our subjects the average weight might be very materially increased.

But in looking over the returns, I find no such case. Out of the 89 cases tabulated, 8 weighed 3 lbs., and only 2 were as high as 3¾ lbs.

On the other hand, 8 weighed about 1½ lbs., 2 were 1¼ lbs., while the lowest weight recorded is 1 case which only weighed 1 lb.

Taking the average weight at 2 lbs. (32 ozs.), the variations noted above are not excessive, and would negative the idea that one or two hypertrophied spleens had vitiated the average.

4. *Pancreas.* This gland is known to atrophy greatly in many aged horses. The College figures show it to be nearly 2½ ozs. lighter in the aged than the adult.

5. *Kidneys.* In the old horse the right kidney weighs 2½ ozs., the left 2¾ ozs., less than in the adult. This would seem to be the result of age. And the atrophy evidently affects both to the same degree.

6. *Brain.* This organ almost more than any other shows the effect of age. I am not aware of any figures giving the proportionate loss in the horse, but the brain of man loses about one ounce in weight for every ten years after the age of forty. We would reasonably expect then to find the brain lighter in the subjects from which our figures are taken; instead of this, we find the aged brain to be heavier than that of the adult by about one-third of an ounce. I shall not attempt any explanation of the figures.

Speaking generally our figures tend to confirm those of Chauveau, and also to illustrate the effect of age upon the various organs.

